# Supplementary material for: The genome sequence of the yellow-legged black legionnaire, Beris morrisii (Dale, 1841)
Source: Wellcome Open Res. 2023 Nov 15;8:527. [Version 1] doi: 10.12688/wellcomeopenres.20352.1 (PMC11128770; doi:10.12688/wellcomeopenres.20352.1)
Supplement: Peer review history

# Reviewer report: Reviewer response for version 1

**DOI:** 10.21956/wellcomeopenres.22537.r80427

**Published:** 2024-05-26

**Authors:** Levente Laczkó

**Affiliations:** 1 University of Debrecen, Debrecen,, Hungary

In this paper, the authors present the high-quality genome assembly of Beris morrisii, in which multiple sequencing approaches were used to reconstruct the complete genome of the species. The background is well described and it is stated that this resource will aid research into the species, which I agree with. The sequencing and data analysis methods are appropriate. The primary assembly has high contiguity and the assembly was finalized using Hi-C contact maps, with potential contaminants also identified. The final assembly has a high completeness. The methodology is detailed and reproducible. The metadata of the samples are very well described, which facilitates the reusability of the data. I have no concerns about this article and support its publication

Are sufficient details of methods and materials provided to allow replication by others?

Yes

Is the rationale for creating the dataset(s) clearly described?

Yes

Are the datasets clearly presented in a useable and accessible format?

Yes

Are the protocols appropriate and is the work technically sound?

Yes

Reviewer Expertise:

genomics, bioinformatics

I confirm that I have read this submission and believe that I have an appropriate level of expertise to confirm that it is of an acceptable scientific standard.

# Reviewer report: Reviewer response for version 1

**DOI:** 10.21956/wellcomeopenres.22537.r80462

**Published:** 2024-05-02

**Authors:** Jullien M Flynn

**Affiliations:** 1 Whitehead Institute for Biomedical Research, Cambridge, MA,, USA

In this article, the authors present a genome assembly for *Beris morrisii. *The assembly methods are well-described and the data are well-presented. The data, detailed protocols, and the assembly are publicly available. The Hi-C contact maps show an interesting pattern of interchromosomal contacts, which may represent contacts between regions such as the centromeres. I have no concerns about this article.

Are sufficient details of methods and materials provided to allow replication by others?

Yes

Is the rationale for creating the dataset(s) clearly described?

Yes

Are the datasets clearly presented in a useable and accessible format?

Yes

Are the protocols appropriate and is the work technically sound?

Yes

Reviewer Expertise:

genomics, repetitive DNA, evolution

I confirm that I have read this submission and believe that I have an appropriate level of expertise to confirm that it is of an acceptable scientific standard.
